# Biomechanical Effects of Cement Augmentation and Prophylactic Vertebroplasty on Adjacent Segment Stability in Multilevel Spinal Fusion: A Finite Element Analysis

**DOI:** 10.3390/bioengineering12101071

**Published:** 2025-10-01

**Authors:** Jae Won Shin, Dae Hyeon Kim, Ki Mun Kang, Tae Hyun Park, Yu Rim Oh, Sung Jae Lee, Byung Ho Lee

**Affiliations:** 1 Department of Orthopedic Surgery, Yonsei University College of Medicine, Seoul 06273, Republic of Korea; jaewuni11@gmail.com; 2 Department of Biomedical Engineering, College of Biomedical Science & Engineering, Inje University, Gimhae 50834, Republic of Korea; eogus6261@gmail.com (D.H.K.); rkdrlans369@gmail.com (K.M.K.); thyun06@gmail.com (T.H.P.); oyl101526@gmail.com (Y.R.O.)

**Keywords:** spinal fusion, vertebroplasty, bone cements, pedicle screws, range of motion

## Abstract

Background: Multilevel posterior spinal fusion to T10 often encounters complications such as screw loosening and proximal junctional kyphosis. Cement augmentation or prophylactic vertebroplasty is used to prevent these, but their biomechanical effects remain unclear. Methods: A validated finite element model (T8–pelvis) from CT data of a 57-year-old male was tested in five configurations: fusion only, fusion with cement augmentation at T10, T10–T11, T10–T11 plus T9 vertebroplasty, and T10–T11 plus T8–T9 vertebroplasty. Range of motion (ROM), intradiscal pressure (IDP), posterior ligament/facet stress, and cement–bone interface stresses were analyzed under a 400 N follower load and 10 N·m moments. Results: Cement augmentation at the upper instrumented vertebra produced <5% changes in ROM, IDP, and posterior ligament/facet stresses compared with fusion only, indicating preserved stability. Prophylactic vertebroplasty redistributed stress proximally, with elevated cement–bone interface stresses localized at T9 when vertebroplasty was performed at a single adjacent level (T9) and distributed to both T8 and T9 when performed at two adjacent levels (T8–9)—with T9 stressed mainly during lateral bending and extension, and T8 during flexion and lateral bending. Conclusion: Cement augmentation alone did not compromise adjacent-level biomechanics, but prophylactic vertebroplasty created abnormal stress concentrations at adjacent interfaces, potentially increasing fracture risk. These findings highlight the need for careful patient selection and further studies in osteoporotic populations.

## 1. Introduction

Multilevel spinal fusion is a widely performed surgical procedure for the treatment of spinal deformities and degenerative diseases [1,2]. However, when the proximal fusion level extends to the thoracic spine, particularly at T10, mechanical complications such as screw loosening, adjacent segment degeneration, proximal junctional kyphosis (PJK), and, in severe cases, proximal junctional failure (PJF) are frequently observed [3]. These complications negatively affect surgical outcomes and often necessitate revision surgery. A meta-analysis of 14 studies with 2215 patients reported that the incidence of PJK after spinal fusion ranges from 17% to 62%**,** depending on fusion level, patient age, and follow-up duration [4]. To mitigate them, two strategies are commonly employed: cement augmentation of pedicle screws at the uppermost instrumented vertebra (UIV) and prophylactic vertebroplasty of adjacent vertebrae [5,6]. Although these procedures are increasingly used, their true biomechanical efficacy remains controversial. For example, a retrospective study comparing kyphoplasty alone vs. kyphoplasty plus prophylactic vertebroplasty of adjacent vertebrae found that adjacent--segment fractures (ASFs) were significantly more frequent in the vertebroplasty group (50%) compared to the kyphoplasty--only group (16%), suggesting that prophylactic vertebroplasty may not prevent ASFs and might carry additional risk [7].

Finite element modeling (FEM) has become an indispensable method for investigating spinal biomechanics under controlled physiological loading conditions [8]. Previous studies have demonstrated that cement-augmented screws increase fixation strength and reduce screw pullout risk in osteoporotic bone [9], while vertebroplasty enhances vertebral body stiffness and load-bearing capacity [10]. Nevertheless, most prior studies focused on isolated applications of cement augmentation or vertebroplasty, without fully addressing their combined effects in long thoracolumbar fusion. Moreover, clinical series and biomechanical reports suggest that although these techniques may reduce immediate instability, they may also alter stress transfer and increase the risk of adjacent vertebral fractures [11,12,13].

Recent reviews and finite element investigations have emphasized the need to analyze not only the global stability but also the detailed stress redistribution patterns at adjacent levels, especially in high-risk populations with osteopenia or osteoporosis [14,15]. However, despite the growing body of FEM research on spinal fusion and cement augmentation, there remains a paucity of comprehensive analyses addressing the combined role of cement augmentation and prophylactic vertebroplasty in long constructs terminating at the thoracic spine (e.g., T10), where junctional complications are most prevalent. These insights underscore a gap in the literature: the lack of comprehensive FEM studies evaluating both cement augmentation and prophylactic vertebroplasty together in the context of multilevel spinal fusion extending to the thoracic spine.

Recent clinical studies have highlighted the complexity of mechanical complications after long thoracolumbar fusion. For instance, Murata et al. reported that low Hounsfield unit values of the upper instrumented vertebra were significantly associated with postoperative junctional fractures, underscoring the role of bone quality in junctional vulnerability [16]. Similarly, Sawada et al. demonstrated that osteoporotic vertebrae substantially increase the short-term risk of distal or proximal junctional kyphosis following corrective surgery, suggesting that both local bone strength and construct rigidity critically influence outcomes [15]. These findings indicate that cement augmentation or prophylactic vertebroplasty should not be regarded as purely mechanical reinforcements but rather as interventions whose long-term effectiveness is modulated by patient-specific bone characteristics.

Biomechanical investigations have also advanced our understanding of how cement alters load transfer in fused spines. Polikeit et al. showed through finite element analysis that cement augmentation in osteoporotic functional spinal units can substantially modify stress distributions, potentially increasing adjacent-level loading [11]. Chen et al. further demonstrated that higher cement volumes may exacerbate stress concentration at neighboring vertebrae, suggesting a dose-dependent effect [13]. More recently, Meng et al. reported that intradiscal cement leakage after kyphoplasty significantly elevated adjacent vertebral stress, thereby heightening fracture risk [17]. Collectively, these biomechanical findings underscore the delicate balance between enhancing immediate fixation and inadvertently predisposing patients to new adjacent fractures, emphasizing the clinical importance of refining augmentation strategies.

The present study addresses this gap by employing a validated finite element model (T8–pelvis) to investigate the biomechanical effects of cement augmentation and prophylactic vertebroplasty under various surgical configurations. Specifically, we analyzed changes in range of motion, intradiscal pressure, posterior ligament and facet stresses, and cement–bone interface stresses, thereby providing new evidence to guide surgical decision-making and to minimize complications associated with long thoracolumbar fusion.

## 2. Materials and Methods

### 2.1. Ethical Considerations

This study was approved by the Institutional Review Board of Yonsei University College of Medicine (IRB No. 4-2020-0060, approved in January 2020). All procedures were conducted in compliance with the Declaration of Helsinki and the institutional guidelines of our university. Informed consent was obtained from the participants prior to study commencement.

### 2.2. Finite Element Analysis of an Intact Model

Computed tomography data from a healthy 57-year-old male with normal bone (spine bone mineral density T-score = 0.1, measured using dual-energy X-ray absorptiometry), captured at 2-mm intervals, were used to reconstruct the T8–T12 vertebrae using Mimics software (version 24.0; Materialise, Leuven, Belgium). An intact T8-pelvis FE model was constructed by incorporating a previously validated thoracolumbosacral-pelvic model (L9-pelvis) [18]. All models applied 0.3 mm tetrahedral elements (C3D4) through a mesh convergence study to reduce the error of finite element analysis. The thoracic, lumbar, sacral, and pelvic bones were subdivided into cortical and cancellous bone components. Additionally, posterior elements, including the spinous processes and facet joints, were modeled. The posterior complex ligaments of the spine and pelvic ligaments were implemented using the Wire function in ABAQUS software (version 6.24, Dassault Systèmes, Vélizy-Villacoublay, France). Material properties such as Young’s modulus, Poisson’s ratio, and stiffness coefficients were assigned to each bone component based on previously published studies (Supplementary Tables S1 and S2 and Supplementary Figure S1) [19].

### 2.3. Finite Element Model Verification

The FE model was validated to improve its predictive accuracy. Due to the absence of whole-body spine validation data in previous studies, we validated the thoracic, lumbar, sacral, and pelvic regions separately. A pure moment of 7.5 N·m [12,20,21] and 10 N·m was applied to the thoracic and lumbar models, respectively. For the sacral-pelvic model, a 42 N·m pure moment and 294 N translational force were applied based on physiological loading conditions [19,22]. The overall realism of the full FE model was indirectly validated by comparing its predicted responses with experimental data for each region (Supplementary Figures S2–S4).

### 2.4. Finite Element Analysis of Surgical Models

To construct surgical models, oblique lateral interbody fusion and posterior lumbar interbody fusion cages (GS Medical, Osong, Republic of Korea) were converted into finite element models using commercial software SolidWorks (SolidWorks 2024, Dassault Systèmes) (Supplementary Figure S5).

The discs of each intact lumbar segment were removed, oblique lateral interbody fusion cages were inserted at L1–L5, and a posterior lumbar interbody fusion cage was inserted at L5–S1. For posterior fixation, the ANYPLUS Pedicle Screw System (GS Medical) was used. Screws with a diameter of 6.5 mm and a length of 45 mm were applied to the vertebrae, while screws with a diameter of 8.5 mm and a length of 80 mm were applied to the ilium. The pedicle screws were inserted at a 70° angle relative to the transverse plane, targeting the midpoint of the pedicle [23]. Subsequently, 5.5 mm-diameter rods were designed and fixed according to the position of the pedicle screw housings (Supplementary Figure S6). All implants were fabricated from Ti6Al4V, with the material properties (Young’s modulus: 110,000 MPa, Poisson’s ratio: 0.35) applied to the analysis [24].

To apply cement augmentation, previous studies were referenced, and a circular distribution pattern of cement was selected. A surgical model was constructed by designating the anterior 2/3 as the target for cement injection, simulating clinical observations of cement application (Supplementary Figure S7). The cement volumes for both cement augmentation and vertebroplasty averaged 4.037 cc, with values ranging from 4.023 cc to 4.122 cc, depending on the segment (Supplementary Table S3). This value was determined based on prior biomechanical studies indicating that approximately 3–4 mL of cement is generally sufficient to restore vertebral body stiffness in thoracolumbar segments [25]. Additionally, the volume was adjusted according to the anatomical characteristics of the thoracic vertebrae, which have smaller vertebral body volumes compared to lumbar levels. Therefore, the final injection volume reflected a balance between biomechanical reinforcement and the anatomical capacity of each vertebral level [12]. The material properties of polymethylmethacrylate were applied to the bone cement (Supplementary Table S4) [25,26].

**Figure 1:**
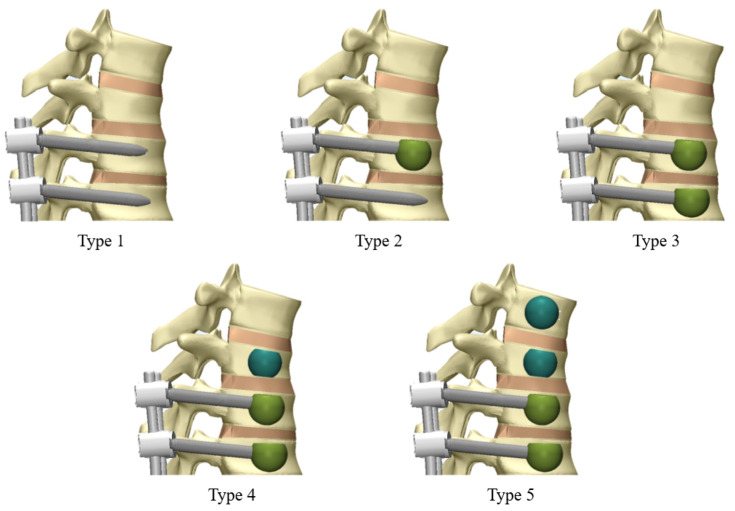
Construction of five different surgical model types (T8 to Pelvis model): Type 1, no cement; Type 2, T10 cement augmentation; Type 3, T10–11 cement augmentation; Type 4, T10–11 cement augmentation with T9 vertebroplasty; and Type 5, T10–11 cement augmentation with T8–9 vertebroplasty.

Five surgical models were developed to simulate varying scenarios of multilevel spinal fusion (Figure 1):

1. Type 1: Fusion with pedicle screws only.
2. Type 2: Fusion with pedicle screws and cement augmentation at T10.
3. Type 3: Fusion with pedicle screws and cement augmentation at T10 and T11.
4. Type 4: Fusion with pedicle screws, cement augmentation at T10 and T11, and vertebroplasty at T9.
5. Type 5: Fusion with pedicle screws, cement augmentation at T10 and T11, and vertebroplasty at T8 and T9.

### 2.5. Boundary and Loading Conditions

To simulate physiological conditions, a 400 N follower load was applied to the spinal column to mimic muscle forces, and a pure moment of 10 N·m was applied to the upper endplate of T8 to simulate four principal motions: flexion, extension, left axial rotation, and left lateral bending. Both acetabular regions were fixed in all directions to replicate a standing posture. Tie contact conditions were applied at implant-implant and bone-implant interfaces to simulate complete fusion.

### 2.6. Outcome Measures

Key biomechanical parameters assessed included ROM, intradiscal pressure (IDP), peak von Mises stress (PVMS), and stress distribution. ROM was measured at T8–T9 and T9–T10 to evaluate structural stability. IDP was assessed at the same levels to predict the potential for adjacent segment degeneration. PVMS was calculated for the posterior ligament complex and facet joints to estimate stress concentrations. Stress distribution was examined across the vertebral bodies to assess potential stress-shielding effects induced by cement augmentation. Differences in these parameters between models were considered negligible if they were within 5% of the corresponding value in the fusion-only model; therefore, small variations fell within the expected numerical error margin of our finite element analysis [8].

## 3. Results

### 3.1. ROM at T8–T9 and T9–T10

**Figure 2:**
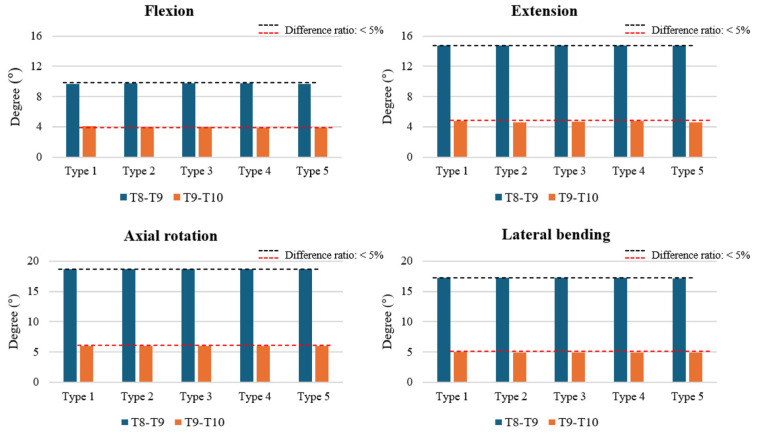
Range of motion (ROM) at the T8–T9 and T9–T10 segments across various surgical models. The ROM values are presented in degrees for flexion, extension, axial rotation, and lateral bending, demonstrating the impact of cement augmentation and vertebroplasty on spinal segment mobility. Notably, no significant differences in ROM were observed across the different surgical types, with all values remaining within the error margin of <5%.

The ROM at T8–T9 and T9–T10 was analyzed across all models. At both levels, the differences between the fusion-only model (Type 1), cement-augmented models (Types 2 and 3), and cement augmented with vertebroplasty models (Types 4 and 5) were less than 5% in flexion, extension, axial rotation, and lateral bending. These findings indicate that neither cement augmentation nor prophylactic vertebroplasty had a meaningful effect on segmental motion at T8–T9 or T9–T10. (Figure 2).

### 3.2. IDP at T8–T9 and T9–T10

**Figure 3:**
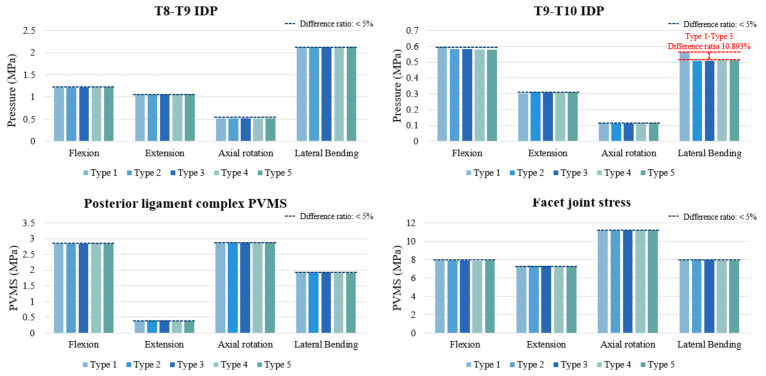
Intradiscal pressure (IDP) at the T8–T9 and T9–T10 segments, peak von Mises stress (PVMS) in the posterior ligament complex, and facet joint stress at T8–T9 under all motion types (flexion, extension, axial rotation, and lateral bending). Most values across surgical models remained within a 5% variation threshold, except for a notable increase in T9–T10 IDP during lateral bending in the Type 1 model.

The IDP at T8–T9 and T9–T10 was evaluated across all models. Overall, the differences among models were within 5% under flexion, extension, and axial rotation, indicating negligible impact of cement augmentation or vertebroplasty on disc loading. At T8–T9, IDP values ranged narrowly across models, with no meaningful differences. At T9–T10, IDP values were also similar across most loading modes. However, during lateral bending, the fusion-only model (Type 1) exhibited an approximately 10.9% higher IDP compared with the cement-augmented and vertebroplasty models (Types 2–5). Despite this relative difference, the absolute magnitude of IDP change was small (~0.05 MPa) and within physiological limits (Figure 3).

### 3.3. PVMS in the PLC and Facet Joint Stress (MPa) at T8–T9

The PVMS in the PLC and facet joints at T8–T9 showed minimal variation across all models and loading directions. In flexion, extension, axial rotation, and lateral bending, differences between the fusion-only, cement-augmented, and vertebroplasty models were within 5%. Facet joint stress patterns closely mirrored those of the PLC, with nearly identical values across all motions. These findings indicate that neither cement augmentation nor prophylactic vertebroplasty meaningfully altered posterior element loading at the uppermost instrumented level (Figure 3).

### 3.4. Cement–Bone Interface Stress Distribution at Adjacent Levels (T8 and T9) During Flexion, Extension, Axial Rotation, and Lateral Bending

**Figure 4:**
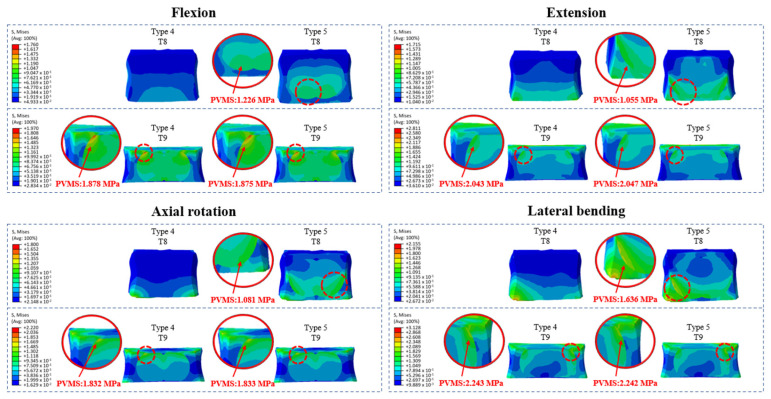
Cement–bone interface stress distribution (MPa) at T8 and T9 during flexion, extension, axial rotation, and lateral bending in Type 4 (T10–11 cement augmentation with T9 vertebroplasty) and Type 5 (T10–11 cement augmentation with T8–9 vertebroplasty) models. Areas with elevated stress levels are highlighted and magnified to show the specific distribution and magnitude.

**Figure 5:**
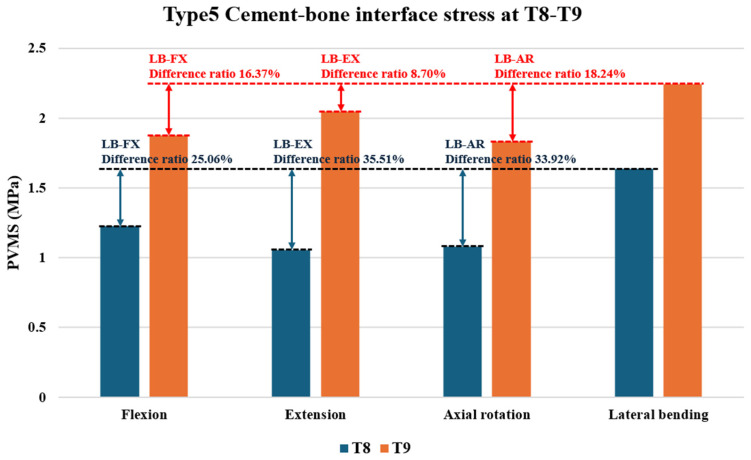
Peak von Mises Stress (PVMS) (MPa) at T8 and T9 in Type 5 (T10–11 cement augmentation with T8–9 vertebroplasty) during flexion, extension, axial rotation, and lateral bending. Lateral bending served as the reference point for error margin calculations, with other motions compared against it.

Cement augmentation alone (Types 2 and 3) did not significantly alter stress distribution at adjacent levels (T8 and T9) compared to that with fusion alone (Type 1). Additionally, no high-stress concentrations were observed at the cement–bone interface of the augmented vertebrae in Type 2 (T10) or Type 3 (T10 and T11), indicating that uppermost instrumented vertebra (UIV) cement augmentation did not introduce abnormal interface stress. However, prophylactic vertebroplasty (Types 4 and 5) led to significant stress redistribution at the cement–bone interface, particularly in lateral bending and extension. In Type 4, the highest stress was observed at the T9 upper region in lateral bending (2.243 MPa), followed by extension (2.043 MPa, 8.9% lower). In Type 5, the T9 upper region also showed the highest stress in lateral bending (2.242 MPa) and the second highest in extension (2.047 MPa, 8.7% lower). Additionally, the T8 lower region in Type 5 exhibited the highest stress in lateral bending (1.636 MPa) and the second highest in flexion (1.226 MPa, 25.0% lower) (Figure 4 and Figure 5, Supplementary Figure S8).

These findings suggest that prophylactic vertebroplasty significantly increases localized stress, particularly during a specific motion, heightening the risk of adjacent segment fractures, especially at the cement–bone interface.

## 4. Discussion

This study investigated the biomechanical effects of cement augmentation and prophylactic vertebroplasty in multilevel posterior spinal fusion extending to T10. Our findings demonstrate that while both techniques influence stress distribution at the cement–bone interface, the addition of vertebroplasty produced motion-specific stress concentrations at adjacent levels, particularly at T8 and T9. These results are consistent with prior reports highlighting altered load transfer and the potential for stress redistribution following cement augmentation [10,11,27]. By identifying stress redistribution patterns at the thoracic UIV region, our study underscores the trade-off between enhancing immediate fixation strength and increasing long-term biomechanical vulnerability.

Cement augmentation and vertebroplasty did not substantially alter the global stability of the posterior elements. However, the motion-specific stress redistribution observed at T8 and T9 with prophylactic vertebroplasty suggests a potential predisposition to adjacent fractures and junctional complications, particularly in patients with osteoporosis or advanced thoracolumbar kyphosis. These findings highlight the need to balance the short-term benefits of reinforcement with the potential for long-term biomechanical risks [3,14,28,29,30].

To address these risks, recent efforts have focused on biological enhancements, not only at the UIV but also at the adjacent levels (UIV + 1 and UIV + 2). For example, transpedicular injection of recombinant human bone morphogenetic protein-2 with β-tricalcium phosphate at the UIV has been shown to reduce PJK and PJF by improving local bone quality and enhancing screw fixation strength. Although vertebroplasty at UIV + 1 and UIV + 2 increased stress concentration at the cement–bone interface in our study, it is conceivable that biologically mediated bone remodeling strategies might provide a more physiological means of reinforcing these levels without inducing abnormal load transfer. Thus, future work should consider whether biologic augmentation techniques can be extended to adjacent vertebrae to mitigate the risks identified in this study [31].

The clinical relevance of these findings lies in optimizing the use of cement augmentation and prophylactic vertebroplasty to balance structural reinforcement and mitigate fracture risk. While prophylactic vertebroplasty at adjacent levels may enhance immediate fixation, it can also create abnormal bone-interface stress distributions, potentially increasing the likelihood of adjacent-level fractures [5]. This risk is particularly concerning in patients with osteoporosis or advanced thoracolumbar kyphosis, where uneven stress propagation could have significant consequences [16,32]. Our FE model was based on a single middle-aged male with normal bone (T-score = 0.1), which may limit its generalizability to patients with more severe bone loss. Prior studies have shown that in osteoporotic spines, cement augmentation can increase stress and intradiscal pressure at adjacent levels, increasing fracture risk [19]. While our model reflects normal bone quality rather than osteoporosis, it still allows the estimation of stress patterns under compromised bone quality. Further studies using patient-specific models with advanced osteoporosis are needed to validate and expand on these findings. Furthermore, exploring alternative techniques such as dynamic stabilization or less rigid fixation systems [6] may offer valuable insights into reducing stress concentrations and improving long-term outcomes in multilevel spinal fusion.

Despite the insights gained, this study has a few limitations related to modeling simplifications. First, a 400 N follower load was used to simulate the overall muscle support, which, while providing a physiological compressive preload, does not replicate the complex, dynamic activation of spinal musculature. To improve physiological realism, posterior element ligaments were also incorporated into the model to account for passive structural support. However, while these additions improve the model’s fidelity, they may still not fully capture the intricate neuromuscular interactions observed in vivo. Second, we rigidly fixed both iliac regions in the model; this boundary condition may over-constrain the spine compared to in vivo conditions, where the sacroiliac joints allow slight motion. These simplifications could affect the absolute magnitudes of calculated stresses and motions. However, because they were applied uniformly across all model variants, the comparative trends and resulting conclusions are considered valid [14]. This finite element study focused on the mechanical aspects of cement augmentation and prophylactic vertebroplasty in multilevel fusion. Biocompatibility and structural analyses of the alloys were beyond the scope of this work, although they are indeed important factors influencing implant longevity and clinical outcomes. Future studies integrating material-level evaluations with patient-specific finite element models may provide more comprehensive insights.

The clinical implications of these findings are particularly relevant in the context of long thoracolumbar fusion surgeries. The identification of motion-specific stress concentrations at UIV + 1 and UIV + 2 following prophylactic vertebroplasty suggests that such interventions, although intended to reduce fracture risk, may paradoxically increase localized biomechanical vulnerability. This highlights the need to carefully weigh the benefits of immediate reinforcement against the long-term risk of adjacent segment complications. Moreover, our study provides a novel quantitative framework to assess stress propagation patterns across adjacent vertebrae under multilevel fixation constructs—an area that remains underexplored in the current literature. By incorporating a full-length thoracolumbar model and comparing five distinct surgical strategies, this analysis offers new insights into the mechanical behavior of the upper instrumented region and its adjacent segments.

The finite element model was developed to reflect anatomical and biomechanical realism. However, direct validation of the T8–T9 segment was not feasible due to the lack of experimental data matching our simulation. To address this, we compared the simulated range of motion (ROM) at T8–T9 with published values. ROM values were slightly higher than in vitro measurements, likely due to the absence of rib cage stabilization in our model [33,34,35]. Thus, our results are considered reasonable under rib cage-free conditions. Clinically, this approach is relevant for patients undergoing cement augmentation, who often exhibit reduced thoracic stiffness due to osteoporosis or osteopenia. The resulting increase in segmental motion supports the applicability of our model for evaluating spinal fusion strategies in degenerative populations [36]. Another limitation is that the present model employed normal bone material properties, which restricts its applicability to patients with compromised bone quality—a common demographic in multilevel spinal fusion. To enhance clinical relevance and better predict fracture risk, future finite element studies should incorporate osteopenic and osteoporotic bone properties derived from experimental data. In fact, future research is planned to compare models with normal, osteopenic, and osteoporotic bone quality to clarify their differential impact on stress distribution and fracture risk.

## 5. Conclusions

- Cement augmentation at the UIV did not significantly affect ROM, IDP, PLC, or facet joint PVMS, indicating preserved stability.
- UIV cement augmentation alone did not increase stress distribution at adjacent levels.
- Prophylactic vertebroplasty at adjacent levels produced uneven cement–cancellous bone interface stresses, particularly at T8 and T9.
- These abnormal stress concentrations may predispose patients to adjacent-level fractures and contribute to PJK or PJF.
- The results emphasize the trade-off between immediate fixation strength and long-term fracture risk, especially in osteoporotic or kyphotic patients.
- This study provides biomechanical evidence to guide surgical strategies aimed at minimizing complications and improving outcomes in multilevel spinal fusion.
- Future work should focus on osteoporotic, patient-specific finite element models and validation with clinical or experimental data to further refine surgical strategies.

## Data Availability

The original contributions presented in this study are included in the article. Further inquiries can be directed to the corresponding authors.

## Supplementary Materials

The following supporting information can be downloaded at https://www.mdpi.com/article/10.3390/bioengineering12101071/s1: Figure S1: Construction of a three-dimensional spine model using computed tomography data; Figure S2: Comparison of the range of motion (ROM) between the present study and published data under flexion (FX) and extension (EX) motions in the intact T9–L1 and L1–5 segments; Figure S3: Comparison of the range of motion (ROM) between the present study and published data11,12 under axial rotation (AR) and lateral bending (LB) motions in the intact T9–L1 and L1–5 segments; Figure S4: Comparison of the range of motion (ROM) between the present study and published data under sacroiliac joint behavior; Figure S5: Implants used in the study. (A) Size of the pedicle screw (left) and iliac screw (right), (B) Size of the oblique lateral lumbar interbody fusion cage, and (C) Size of the posterior lumbar interbody fusion cage; Figure S6: Surgical configurations. (A) Method of pedicle screw insertion, (B) Method of iliac screw insertion, and (C) Lateral and posterior views of the spine model, highlighting the instrumented levels; Figure S7: Cement application in the finite element model. (A) Distribution of cement within the T8 and T9 vertebral bodies following simulated vertebroplasty. (B) Cement augmentation of pedicle screws at T10 and T11; Table S1: Material properties (ligaments); Table S2: Material properties (bone and soft tissue); Table S3: Vertebral body and cement volume; Table S4: Material properties of bone cement.

## Abbreviations

The following abbreviations are used in this manuscript:

FEM	finite element modeling
IDP	intradiscal pressure
PJF	proximal junctional failure
PJK	proximal junctional kyphosis
ROM	range of motion
UIV	uppermost instrumented vertebra
