# Supplementary material for: Biomechanical Effects of Cement Augmentation and Prophylactic Vertebroplasty on Adjacent Segment Stability in Multilevel Spinal Fusion: A Finite Element Analysis
Source: Bioengineering (Basel). 2025 Oct 1;12(10):1071. doi: 10.3390/bioengineering12101071 (PMC12561517; doi:10.3390/bioengineering12101071)
Supplement: Supplementary file 1 [file bioengineering-12-01071-s001.zip › bioengineering-3866269-supplementary.pdf]

Supplementary Files

Supplementary Table S1. Material properties (ligaments)

**Materials**	**Stiffness coefficient (N/mm)**
Anterior sacroiliac ligament	700
Posterior sacroiliac ligament (long)	1,000
Posterior sacroiliac ligament (short)	400
Interosseous sacroiliac ligament	2,800
Sacrospinous ligament	1,400
Sacrotuberous ligament	1,500
Superior pubic ligament	500
Arcuate pubic ligament	500
Inguinal ligament	250
Iliolumbar ligament	1,000

Stiffness coefficients (N/mm) for ligaments associated with the spine and pelvis.

Supplementary Table S2. Material properties (bone and soft tissue)

Materials	Young's modulus (MPa)	Poisson's ratio
Ilium (cortical)	17,000	0.3
Ilium (cancellous)	132	0.2
Sacrum (cortical)	6,140	0.3
Sacrum (cancellous)	1,400	0.3
Vertebral body (cortical)	12,000	0.3
Vertebral body (cancellous)	100	0.3
Posterior elements	3,500	0.25
Annulus fiber	450	0.3<br>.3
Annulus matrix	4.2	0.45
Nucleus pulposus	1	0.499
Endplate	100	0.3

Young's modulus (MPa) and Poisson's ratio for cortical and cancellous bone, as well as soft tissues.

Supplementary Table S3. Vertebral body and cement volume

	T8	T9	T10	T11
Vertebral body volume	12,183	11,974	15,727	16,671
Cement volume	4,023	4,079	3,923	4,122
Cement ratio	33%	34%	24%	24%

Vertebral body volumes (mm^3) and corresponding cement volumes (mm^3) for each segment (T8–T11), along with cement-to-bone volume ratios (%). These data illustrate the extent of cement augmentation used in the models.

Supplementary Table S4. Material properties of bone cement

**Materials**	**Young’s modulus (MPa)**	**Poisson’s ratio**
Bone cement	3,500	0.3

Material properties of polymethylmethacrylate bone cement, including Young's modulus and Poisson's ratio.

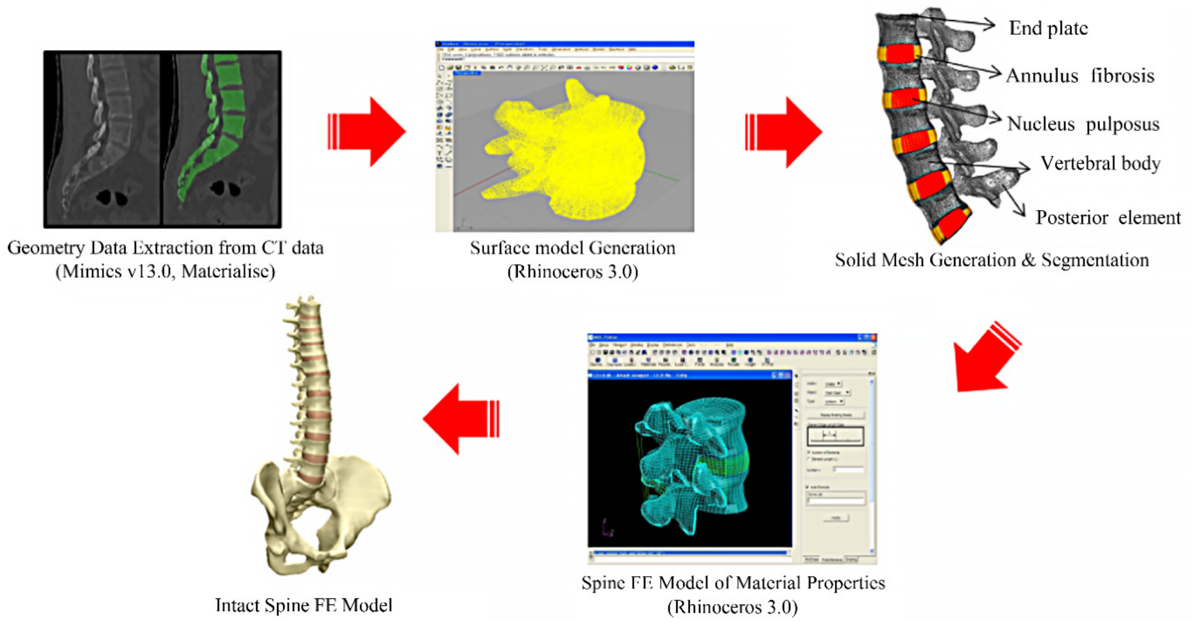

Supplementary Figure S1. Construction of a three-dimensional spine model using computed tomography data

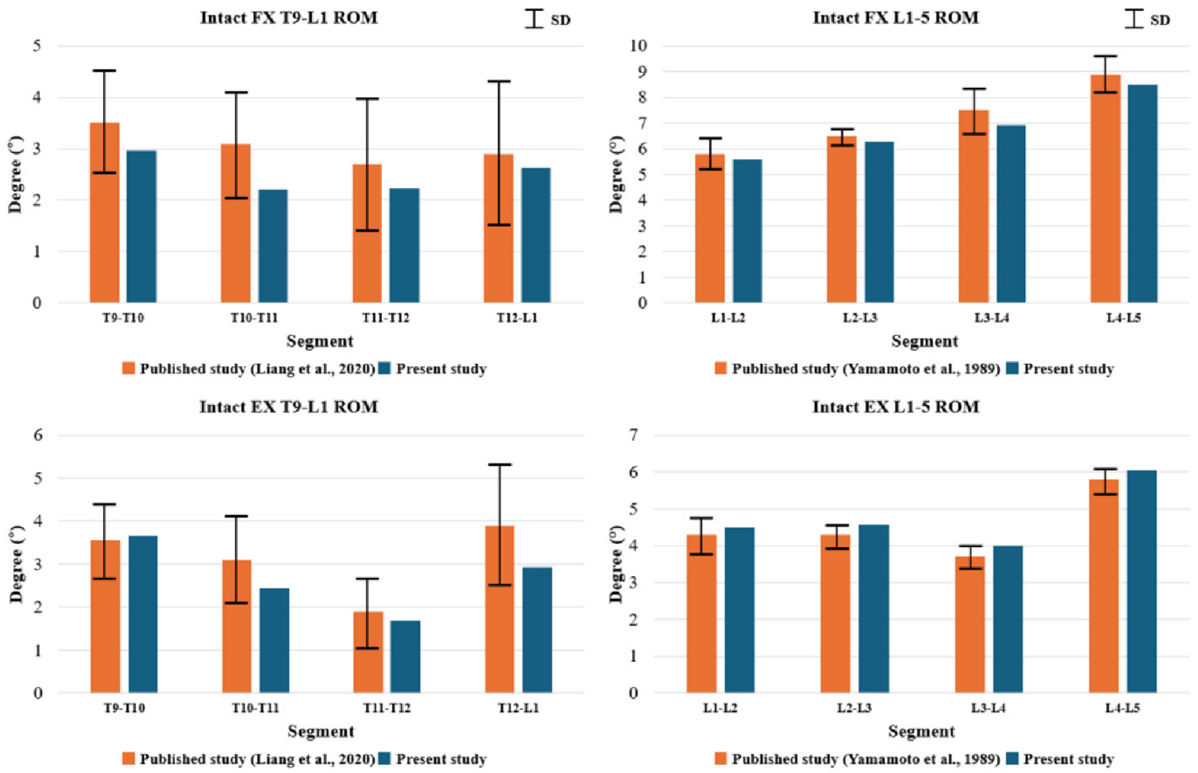

Supplementary Figure S2. Comparison of the range of motion (ROM) between the present study and published data under flexion (FX) and extension (EX) motions in the intact T9–L1 and L1–5 segments[1]; SD, Standard deviation

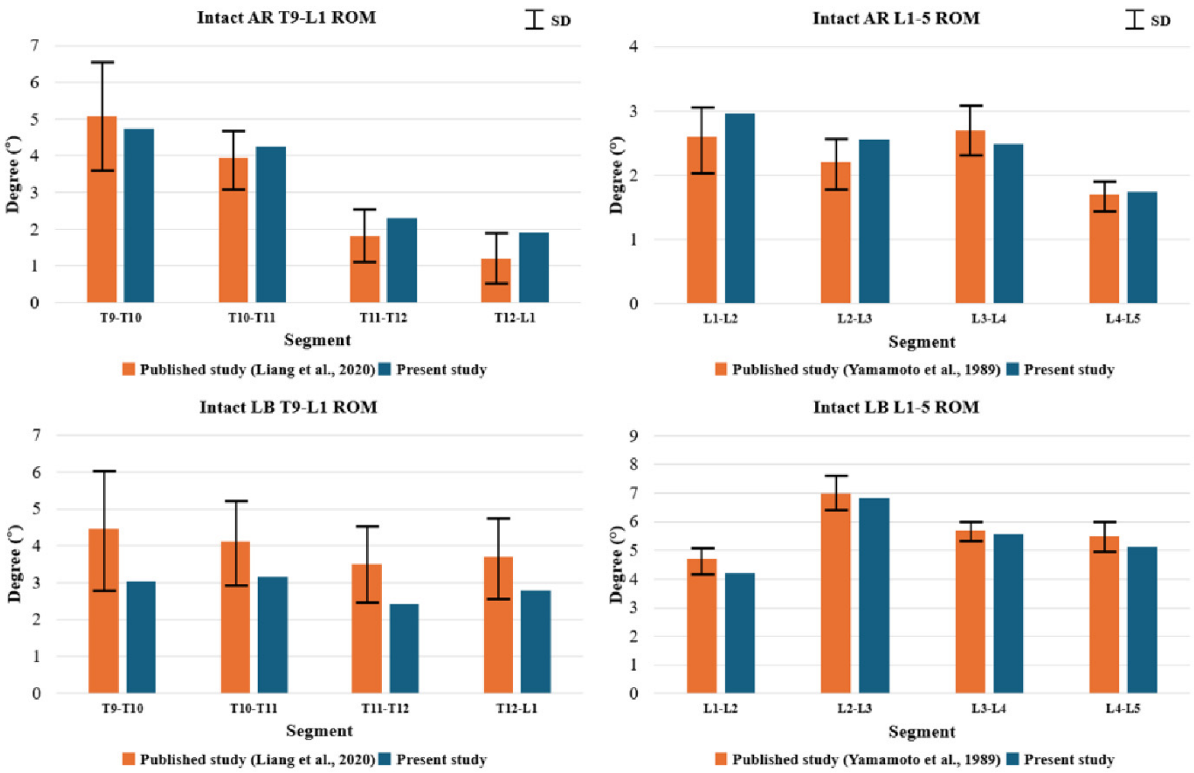

Supplementary Figure S3. Comparison of the range of motion (ROM) between the present study and published data[11,12] under axial rotation (AR) and lateral bending (LB) motions in the intact T9–L1 and L1–5 segments

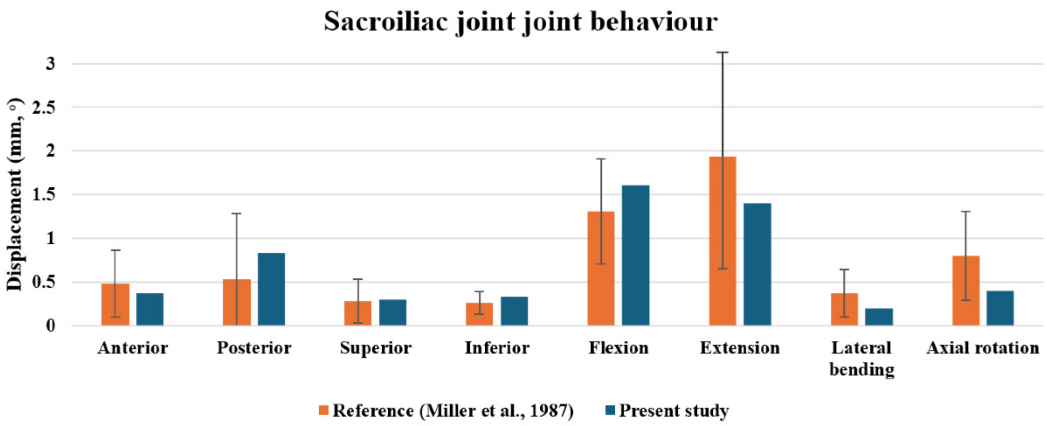

Supplementary Figure S4. Comparison of the range of motion (ROM) between the present study and published data under sacroiliac joint behavior.[14]

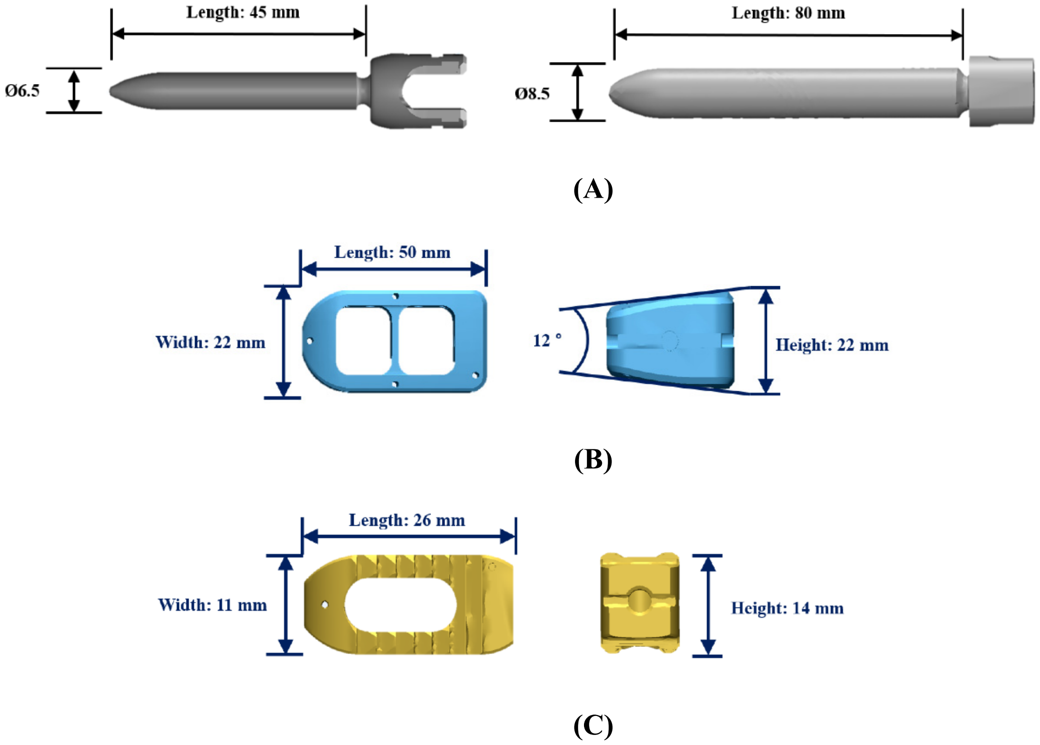

Supplementary Figure S5. Implants used in the study. (**A**) Size of the pedicle screw (left) and iliac screw (right), (**B**) Size of the oblique lateral lumbar interbody fusion cage, and (**C**) Size of the posterior lumbar interbody fusion cage

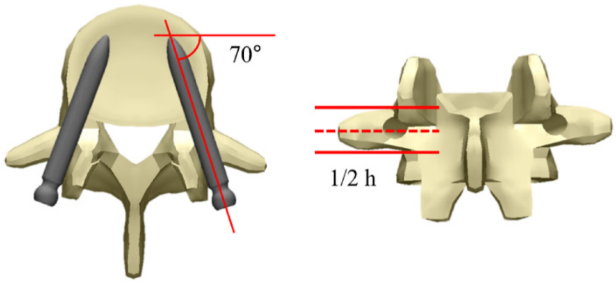

(A)

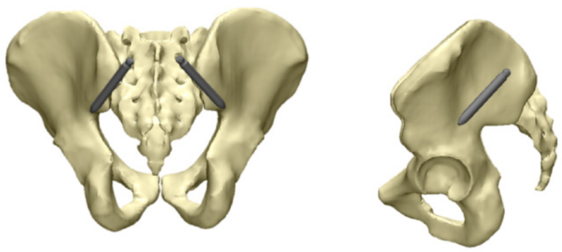

(B)

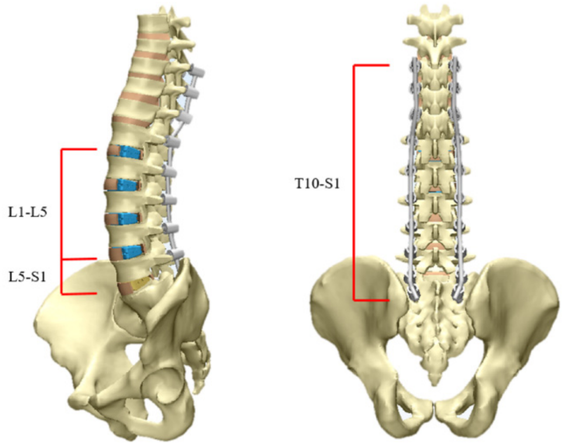

(C)

Supplementary Figure S6. Surgical configurations. (**A**) Method of pedicle screw insertion, (**B**) Method of iliac screw insertion, and (**C**) Lateral and posterior views of the spine model, highlighting the instrumented levels

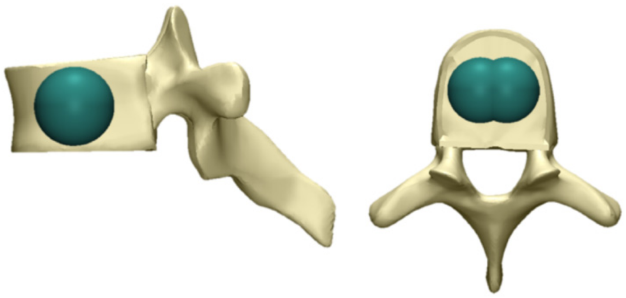

(A)

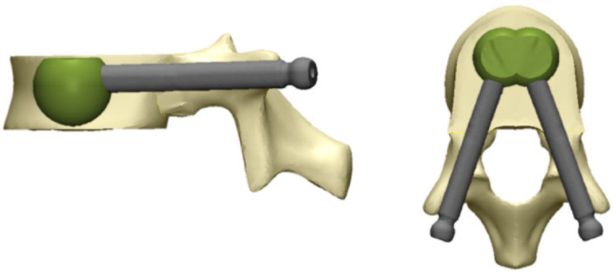

(B)

Supplementary Figure S7. Cement application in the finite element model. (**A**) Distribution of cement within the T8 and T9 vertebral bodies following simulated vertebroplasty. (**B**) Cement augmentation of pedicle screws at T10 and T11.

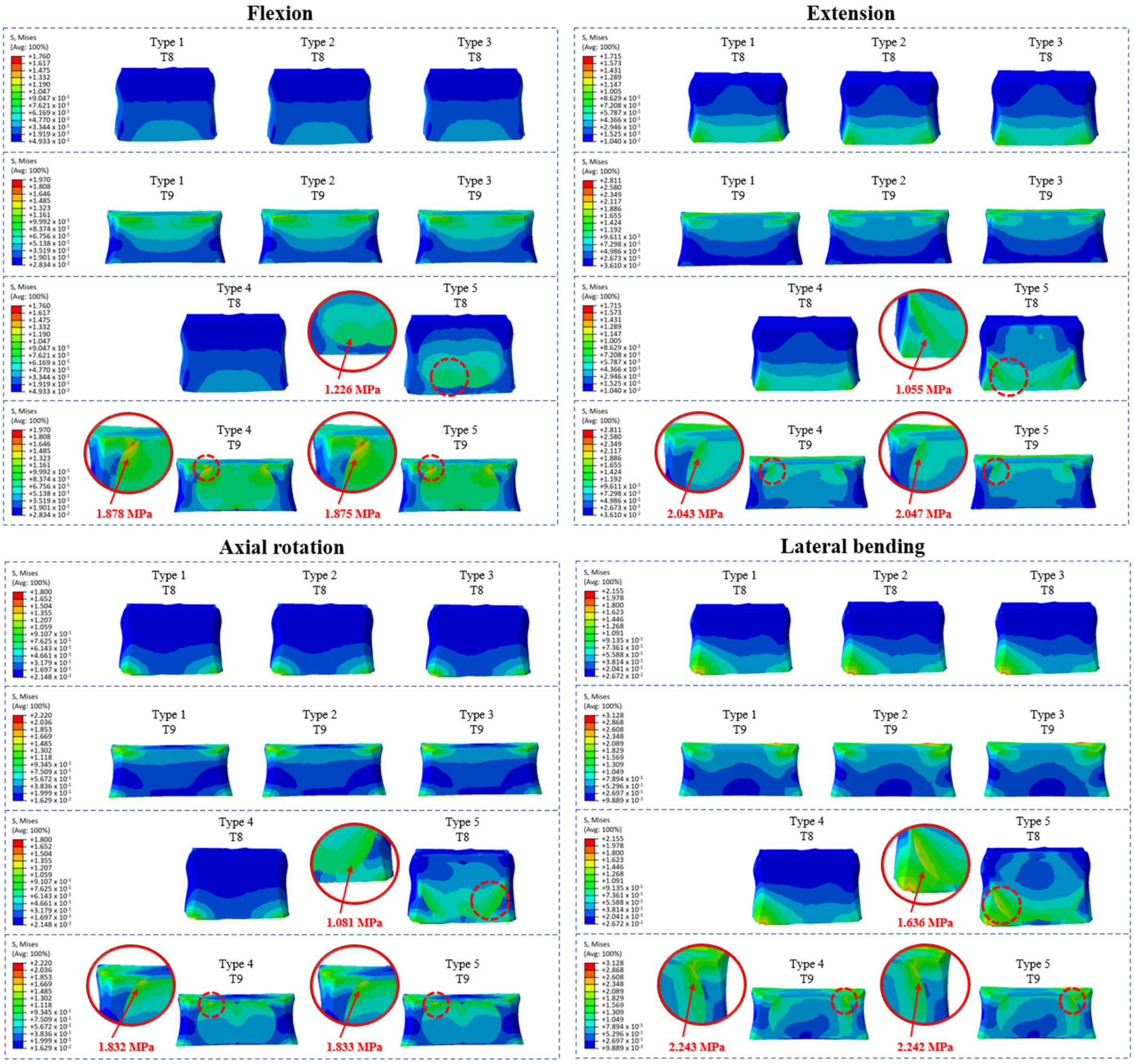

Supplementary Figure S8. Cement-bone interface stress distribution (MPa) at T8 and T9 during flexion, extension, axial rotation, and lateral bending. Areas with elevated stress levels are highlighted and magnified to show the specific stress distribution and magnitude.
